# Synergistic Removal of Diclofenac via Adsorption and Photocatalysis Using a Molecularly Imprinted Core–Shell Photocatalyst

**DOI:** 10.3390/ma18102300

**Published:** 2025-05-15

**Authors:** Ivana Gabelica, Floren Radovanović-Perić, Gordana Matijašić, Kristina Tolić Čop, Lidija Ćurković, Dragana Mutavdžić Pavlović

**Affiliations:** 1 Faculty of Mechanical Engineering and Naval Architecture, University of Zagreb, Ivana Lučića 5, 10000 Zagreb, Croatia; 2 Faculty of Chemical Engineering and Technology, University of Zagreb, Trg Marka Marulića 19, 10000 Zagreb, Croatia; fradovano@fkit.unizg.hr (F.R.-P.); gmatijas@fkit.unizg.hr (G.M.); ktolic@fkit.unizg.hr (K.T.Č.); dmutavdz@fkit.unizg.hr (D.M.P.)

**Keywords:** microwave synthesis, nanocomposites, Fe_3_O_4_/SiO_2_/TiO_2_, magnetic properties, photocatalysis, MIPs, diclofenac

## Abstract

In this work, a newly developed magnetic molecularly imprinted Fe_3_O_4_/SiO_2_/TiO_2_/MIP photocatalyst with diclofenac (DIC) as the template was prepared by microwave-assisted synthesis. The molecularly imprinted TiO_2_ layer has specific cavities designed for the DIC target molecule (imprint), resulting in a synergistic effect of extraction by adsorption and photocatalysis. For reference, non-imprinted magnetic nanoparticles (Fe_3_O_4_/SiO_2_/TiO_2_) were prepared using the same procedure. The obtained particles were characterized by X-ray diffraction analysis (XRD), Fourier transform infrared spectroscopy (FTIR), SEM-EDX, vibrating-sample magnetometry (VSM) and diffuse reflectance spectroscopy (DRS). Specific surface area, pore volume and pore size distribution were evaluated using Brunauer–Emmett–Teller (BET) adsorption–desorption isotherms. The synergistic effect of adsorption and photocatalysis as well as the kinetics and mechanism of DIC degradation using Fe_3_O_4_/SiO_2_/TiO_2_/MIP and Fe_3_O_4_/SiO_2_/TiO_2_ were determined and analysed. The adsorption efficiency of Fe_3_O_4_/SiO_2_/TiO_2_/MIP for DIC (10 mg dm^−3^) was around 86% after 60 min. The DIC (10 mg dm^−3^) removal efficiency of Fe_3_O_4_/SiO_2_/TiO_2_/MIP was around 80% after 30 min adsorption and 120 min of reaction under both UV-A- and solar-simulated light irradiation.

## 1. Introduction

In recent years, numerous studies have focused on the removal of pharmaceutically active compounds and other organic micropollutants (OMPs) from wastewater due to their persistence and continuous release into the aquatic environment [1]. Pharmaceuticals have become one of the most prominent groups of contaminants, owing to an increase in their consumption globally. They involve various substances such as antibiotics, analgesics, antimicrobials, anti-inflammatory drugs, beta blockers and others [2]. However, conventional wastewater treatment plants (WWTPs) are unable to completely remove these contaminants, as their occurrence is observed in the influents and effluents of WWTPs [3,4]. These substances are considered potentially hazardous due to their detrimental effects on ecosystems and their harmful impacts on living organisms [5].

Diclofenac (DIC), 2-(2,6-dichloranilino) phenylacetic acid, is a non-steroidal anti-inflammatory drug used worldwide as an analgesic for arthritis or acute and chronic pain in humans and animals [6]. DIC is also the active ingredient in the well-known Voltaren©, which is used as a gel for short-term pain relief and the treatment of muscle and joint injuries, swelling of tendons, elbows or knees in adults and children, as well as in tablet form for the symptomatic, short-term treatment of pain related to inflammatory ear, throat or nose infections [7]. The worldwide consumption of diclofenac is constantly growing according to the European Geosciences Union General Assembly 2018, with about 2400 tons used annually [8]. These high consumption levels make diclofenac one of the most commonly detected pollutants in both water and soil. Its low biodegradability and high persistence cause cytotoxicity to liver, kidney and gill cells [7,9]. Yu et al. investigated the occurrence, ecological risks and toxicity of diclofenac and concluded that it has adverse effects on the aquatic environment as well as human and animal health, even at low concentrations [10]. They also indicated the synergistic interactions of diclofenac with other contaminants, which lead to the development of new pollutants. In view of all this, it is necessary to develop efficient methods for the complete removal of diclofenac from the aquatic environment.

One of the most promising solutions is heterogeneous photocatalysis, as a part of advanced oxidation processes (AOPs). The generation of highly reactive radicals can be harnessed for oxidative degradation of contaminants in drinking water or wastewater. The most commonly used photocatalyst is TiO_2_, offering several advantages such as low cost, stability, low toxicity, corrosion resistance and availability [11]. The photocatalytic properties of TiO_2_ arise from the generation of charge carriers (holes and electrons) when it absorbs ultraviolet (UV) light, with an energy equal to its band gap (3.2 eV for anatase) [12,13,14]. However, the use of TiO_2_ has some disadvantages, such as the limited applicability of UV light due to the band gap energy and the low selectivity for the targeted pollutant, as well as the difficulty to separate, recycle and reuse the photocatalyst [6,15].

To address the issue of low selectivity, molecular imprint technology can be employed to increase selectivity [16]. Molecular imprinting comprises a template molecule (pharmaceutical), a functional monomer, a crosslinker, a porogen or solvent, and an initiator [17]. The imprinting process usually begins with the formation of a pre-polymerized monomer–template complex in a porogenic solvent, which acts as a dispersion medium and pore-forming agent, followed by the addition of a crosslinker, whose role is to stabilize the structure of the monomer–template complex. Finally, an initiator is used to initiate the polymerization process [18]. After removal of the template molecule, a specific cavity is formed consistently with the template molecule, thereby enhancing selectivity and recognition for the specific template molecule [19].

To overcome the problem of separation of the photocatalyst after the degradation, magnetic nanoparticles can be used as a support [20]. Among various magnetic materials, Fe_3_O_4_ has already attracted significant interest, owing to its excellent characteristics, such as low toxicity, low cost, and simple preparation. Furthermore, magnetic nanoparticles can be easily separated from a solution by an external magnetic field [21]. Shakeel Zeb et al. developed a biomimetic sensor using magnetic nanoparticles and molecularly imprinted polymer (MIP) [22]. The obtained sensor was successfully applied in commercial and raw milk samples for tetracycline detection. Eylem Turan and Ferat Sahin synthesized highly selective oligo(ethylene glycol) monomethyl ether methacrylate (OEGMA) based molecularly imprinted polymers for carcinogenic mycotoxin Ochratoxin A (OTA) on the surface of magnetic nanoparticles via surface-initiated radical atom transfer polymerization [23]. The results report that MIP nanoparticles showed fast adsorption, large adsorption capacity and high selectivity toward Ochratoxin A. Ferreira Santos et al. developed a magnetic MIP for the selective analysis of glutathione by precipitation [24]. The results indicated good functionality of the developed sensor with a low relative error compared to spectrophotometric techniques. Yun Li et al. prepared a highly selective core– shell MIP on the surface of magnetic nanoparticles for the selective extraction and detection of tadalafil from medicines [25]. The resulting molecularly imprinted magnetic nanoparticles demonstrated excellent properties and were effectively employed for the determination of tadalafil in herbal products for sexual health.

A wide range of methods for synthesizing magnetic nanoparticles have been reported in the literature, with microwave synthesis being one of the promising new methods [26,27,28,29,30]. Microwave radiation enables a fast and uniform heating rate, a rapid nucleation and growth of particles, shortens the reaction time, and enables energy savings, while the magnetite core enables easy separation by an external magnet as well as the possibility for reuse [31,32].

Heterogeneous photocatalysis is a promising advanced oxidation method for the removal of emerging organic micropollutants, such as pharmaceuticals, from wastewater. In most cases of heterogeneous photocatalysis, the photocatalyst is used in the form of a suspension. The main disadvantage of suspended photocatalysts is their separation from the solution after the photocatalytic process. In this work, a magnetic nanostructured core–shell photocatalyst of Fe_3_O_4_/SiO_2_/TiO_2_ was synthesized via microwave-assisted synthesis, followed by the creation of a diclofenac molecular imprint through polymerization. The obtained photocatalyst was used for the adsorption and photocatalytic degradation of diclofenac from a suspension.

## 2. Materials and Methods

### 2.1. Materials

For the synthesis of magnetic nanoparticles (Fe_3_O_4_), iron (III) chloride hexahydrate (FeCl_3_ × 6H_2_O; VWR chemicals, Darmstadt, Germany), iron (II) sulfate heptahydrate (FeSO_4_ × 7H_2_O; Alfa Aesar, Kandel, Germany), sodium hydroxide (NaOH; Gram-mol, d.o.o., Zagreb, Croatia), and deionized water (DI) were used.

For the SiO_2_ coating, SiO_2_ sol was prepared by mixing ethanol (EtOH, 96% p.a.; Gram-mol d.o.o., Zagreb, Croatia) as the solvent and tetraethoxy silane (TEOS; Merck, Darmstadt, Germany) was used as the precursor, while ammonia (NH_3_, 25%; Gram-mol d.o.o., Zagreb, Croatia) was used as the catalyst.

For the TiO_2_ coating, TiO_2_ sol was prepared by mixing titanium(IV) isopropoxide TIP (Sigma-Aldrich, St. Louis, MO, USA) as the precursor; 2-propanol (PrOH; Gram-mol d.o.o., Zagreb, Croatia) was used as the solvent, acetylacetone (AcAc; Sigma-Aldrich, St. Louis, MO, USA) was utilized as the chelating agent, and nitric acid (HNO_3_; Carlo Erba Reagents, Barcelona, Spain) was employed as the catalyst.

For the microwave-assisted polymerization of diclofenac (Fe_3_O_4_/SiO_2_/TiO_2_/MIP), diclofenac sodium salt (DIC, Sigma Aldrich, Buchs, Switzerland) was used as a template molecule, acetonitrile (ACN Sigma Aldrich, Buchs, Switzerland) as a porogen and solvent, methacrylic acid (MAA, >99%, Tokyo Chemical Industry, Tokyo, Japan) as a functional monomer, ethylene glycol dimethacrylate (EGDMA, >97%, Tokyo Chemical Industry, Tokyo, Japan) as a crosslinker, and 2,2′-Azobis(2-methylpropionitrile) (AIBN, 98%, Sigma Aldrich, Buchs, Switzerland) as the initiator.

### 2.2. Microwave-Assisted Synthesis of Magnetic Core–Shell Fe_3_O_4_/SiO_2_/TiO_2_ Nanoparticles

The procedure for the preparation of magnetic nanoparticles was described in detail in our previous work [30]. Briefly, Fe_3_O_4_ nanoparticles were synthesized by reacting FeCl_3_ × 6H_2_O and FeSO_4_ × 7H_2_O with NaOH, followed by microwave irradiation at 100 °C for 5 min and magnetic separation, washing and drying. These particles were coated with SiO_2_ using TEOS in ethanol and ammonia, and then separated, washed and dried. Finally, the Fe_3_O_4_/SiO_2_ particles were added to TiO_2_ sol, which was prepared by mixing Pr-OH, AcAc, TIP, and 0.5 M HNO_3_ in a TIP:PrOH:AcAc:HNO_3_ molar ratio of 1:35:0.63:0.015. The mixture was then microwaved at 200 °C for 10 min to obtain Fe_3_O_4_/SiO_2_/TiO_2_ nanocomposite, which was magnetically separated, washed, and dried. The inner pressure and temperature were monitored during the synthesis process using a *p*/*T* sensor accessory (Anton-Paar GmbH, Graz, Austria), as shown in the Supplementary Material (Figures S1 and S2).

### 2.3. Microwave-Assisted Polymerization of Diclofenac MIP (Fe_3_O_4_/SiO_2_/TiO_2_/MIP)

The template molecule, diclofenac (0.2 mmol), was dissolved in 20 mL of acetonitrile in an Erlenmeyer flask. Methacrylic acid (2.0 mmol), EGDMA (10.0 mmol), AIBN (164 mg) and Fe_3_O_4_/SiO_2_/TiO_2_ nanoparticles (0.25 g) were then added. To obtain a good dispersion of nanoparticles, the solution was sonicated in an ultrasound bath for 5 min and transferred into Teflon tubes equipped with caps. Then, the solution was purged with a gentle flow of nitrogen for 5 min and sealed. Polymerization was carried out in a microwave oven (Anton Paar Microwave Reaction System SOLV, Multiwave PRO, Graz, Austria) following the power-controlled regime: 500 W for 5 min, holding at 500 W for an additional 5 min and then cooling. After the polymerization, the solid particles (Fe_3_O_4_/SiO_2_/TiO_2_/MIP) were collected by centrifugation. The template was removed by washing with methanol/acetic acid solution (9:1, *V*/*V*), until UV spectrometric measurements of the washing solvent showed no detectable template. The inner pressure and temperature were monitored during the synthesis process using a *p*/*T* sensor accessory (Anton-Paar GmbH, Graz, Austria), as shown in the Supplementary Material (Figure S3).

### 2.4. Materials Characterization

Both particles, i.e., Fe_3_O_4_/SiO_2_/TiO_2_ and Fe_3_O_4_/SiO_2_/TiO_2_/MIP, were characterized via FTIR, XRD, vibrating-sample magnetometry (VSM), SEM-EDX and DRS. BET adsorption–desorption isotherms were used to evaluate the specific surface area, pore volume, and pore size distribution.

FTIR measurements were performed using IRSpirit (Shimadzu, Tokyo, Japan) equipped with the single-reflection attenuated total reflectance (ATR) accessory in the wavenumber range of 400 to 4000 cm^−1^.

XRD was performed on XRD-6000 (Shimadzu, Tokyo, Japan) using CuKα radiation at an accelerating voltage of 40 kV and a current of 30 mA. All samples were analysed in a 2θ range of 5–75° in a continuous mode with a 0.02° 2θ step and a scan rate of 0.6 s.

VSM was performed using a Lake Shore Cryotronics vibrating-sample magnetometer (Westerville, OH, USA). Hysteresis measurements were conducted in continuous acquisition mode with a maximum field of 20 kOe and a step size of 20 Oe.

SEM was conducted via SEM Tescan Vega III Easyprobe, with an accelerating voltage of 10 kV, equipped with secondary (SE) and backscattered electron (BSE) detectors (Tescan, Brno, Czech Republic). In addition to SEM, an X-ray (EDX) spectrometer (Bruker B-Quantax, Billerica, MA, USA) was used to analyse chemical composition. The samples were pressed on a carbon tape and measured uncoated at a magnification of 5000 and a scan rate (counts) of 60 × 10^3^ s^−1^.

Diffuse reflectance spectroscopy was used to determine the optical bandgaps of the materials. Diffuse reflectance spectra were collected in a 250 to 1000 nm wavelength range on a Perkin Elmer Lambda 1050+ spectrophotometer (Waltham, MA, USA) using an integrating InGaAs sphere where the sample was rotated by 8° to remove specular reflection. Bandgaps were determined by the slopes of the Tauc plots, which were obtained from the data.

The Brunauer–Emmett–Teller nitrogen adsorption–desorption isotherms were determined at −196 °C using an ASAP-2000 instrument (Micromeritics, Norcross, GA, USA). Prior to measurement, samples were degassed at 150 °C under vacuum until the pressure dropped below 50 mm Hg to remove all adsorbed impurities. The specific surface area (SBET) was calculated using the BET nitrogen adsorption/desorption isotherms based on five data points within the relative pressure range of 0 to 0.2. The pore volume (*V*_p_) and pore size distribution were determined from the nitrogen adsorption isotherm using the Barrett–Joyner–Halenda (BJH) method.

### 2.5. Adsorption, Photocatalysis and Photolysis Evaluation

**Figure 1:**
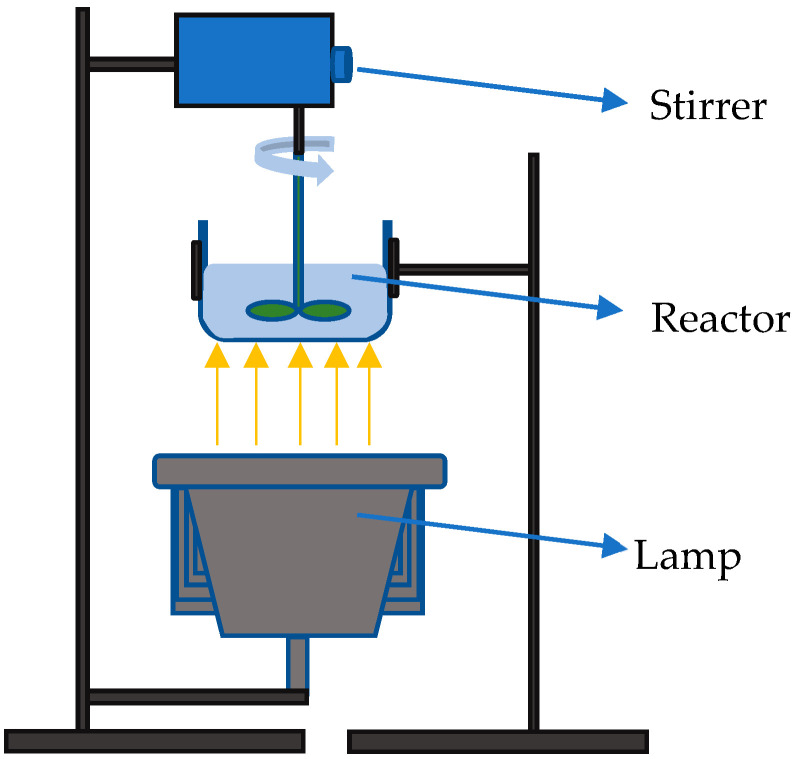
Reactor for photocatalytic experiments.

The adsorption, photolytic and photocatalytic experiments were performed in a flat-bottomed, round borosilicate glass reactor, as shown in Figure 1. Stirring was provided from above with a polyamide stirring bar at 300 rpm. Adsorption tests were conducted by stirring 100 cm^3^ of the aqueous DIC solution (concentration of 5, 7.5, 10 and 12.5 mg dm^−3^) with 50 mg of the photocatalyst in the dark for 120 min. Samples were collected from the reactor at specific intervals (0, 5, 10, 20, 30, 45, 60, 90, and 120 min), filtered through a 0.22 µm polyethersulfone membrane and analyzed using a UV-VIS spectrophotometer (HEWLETT PACKARD, Model HP 8430, Palo Alto, CA, USA) at 273 nm, which corresponds to the maximum absorption peak of DIC. Based on the obtained results, the adsorption kinetics data were analysed using the Lagergren pseudo-first-order model, the Ho’s pseudo-second-order kinetic model, the Weber–Morris intraparticle diffusion model, and the Boyd diffusion model. The Langmuir and Freundlich isotherm models were also used to describe the adsorption mechanism.

The photocatalytic activity of the prepared molecularly imprinted magnetite-based magnetic nanocomposite was examined by the degradation of DIC as the selected micropollutant. Fifty milligrams of the photocatalyst were dispersed in a 100 cm^3^ aqueous DIC solution, with a concentration of 10 mg dm^−3^. The selected lamp was placed under the reactor at a distance of 20 cm and the suspension was irradiated by a UV-A (365 nm) lamp (model UVA HAND LED; Dr. Hönle AG, München, Germany) and solar-simulated lamp (SSL, model SOL500, Dr. Hönle AG, UV-Technologie, Gilching, Germany; 430 W). The global simulated solar radiation was measured by a Kipp & Zonen Co. (Sterling, VA, USA) pyranometer, model CMP11, and the UV-A radiation was measured by a radiometer, model RM 21 with an UV-A Opsytec sensor manufactured by Dr. Gröbel Co. (Ettlingen, Germany). The average global irradiation at a distance of 20 cm from the SSL lamp (at the surface of the reactor) was 1028.6 W/m^2^. The UV-A average irradiation for the SSL was 46.4 W/m^2^, and for the UV-A lamp it was 98.5 W/m^2^. Samples were collected at specific intervals (−30, 0, 5, 10, 20, 30, 45, 60, 90, and 120 min), filtered and analysed using a UV-VIS spectrophotometer.

The photolysis effect was also evaluated by stirring 100 cm^3^ of the aqueous DIC solution (10 mg dm^−3^) under irradiation without the photocatalyst. The reactor setup was the same as for adsorption and photocatalytic measurements (Figure 1). Samples were collected at the same time intervals as for adsorption and filtered and analysed using a UV-VIS spectrophotometer (Analytik Jena GmbH+Co. KG 2025, Spekol 1200, Jena, Germany).

All experiments were performed in triplicate, maintaining less than 5% variation to ensure the reproducibility of the process.

**Figure 2:**
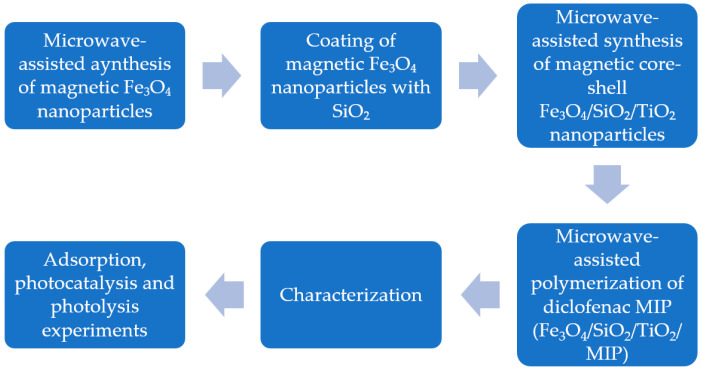
Experimental flowchart.

Figure 2 presents a visual representation of the experimental workflow.

### 2.6. Photocatalytic Mechanisms

To determine the photocatalytic mechanisms, experiments, same as those mentioned above, were conducted but with the addition of scavengers, i.e., interfering agents (Table 1). The scavengers used were as follows: isopropanol (Gram-mol, Zagreb, Croatia), formic acid (Lach-ner s.r.r., Neratovice, Check Republic), *p*-benzoquinone (Merck KGaA, Darmstadt, Germany) and sodium azide (Kemika, Zagreb, Croatia). Adequate amounts of scavengers (isopropanol, formic acid, *p*-benzoquinone, and sodium azide) were added to a DIC water solution (10 mg dm^−3^) to obtain concentrations of 0.1 M for IPA, formic acid and sodium azide, and 0.1 mM for p-BQ. Then, 50mg of Fe_3_O_4_/SiO_2_/TiO_2_ and Fe_3_O_4_/SiO_2_/TiO_2_/MIP photocatalysts were added to the prepared solution of DIC and scavengers and irradiated under UV- and solar-simulated light (SSL). The filtered samples were taken at the same time intervals as for photocatalytic studies (0, 5, 10, 20, 30, 45, 60 and 120 min) and analysed using high-performance liquid chromatography (HPLC) on the Nexera 40 Series Shimadzu HPLC-PDA system, Kyoto, Japan. The separation of diclofenac was performed on a Kinetex C18 column (Phenomenex, Torrance, CA, USA), dimensions 150 mm × 4.6 mm; 5 µm in isocratic mode for 10 min where the mobile phases consisted of 35% of 0.1% formic acid in Milli-Q water (eluent A) and 65% of 0.1% formic acid in acetonitrile (eluent B). The injection volume was 20 µL while the flow rate was 0.5 cm^3^ min^−1^. Diclofenac was detected based on absorption spectra with a maximal absorption wavelength at 276 nm and retention time of 5.4 min.

## 3. Results and Discussion

### 3.1. Characterisation of Core–Shell Fe_3_O_4_/SiO_2_/TiO_2_ and Fe_3_O_4_/SiO_2_/TiO_2_/MIP Nanoparticles

**Figure 3:**
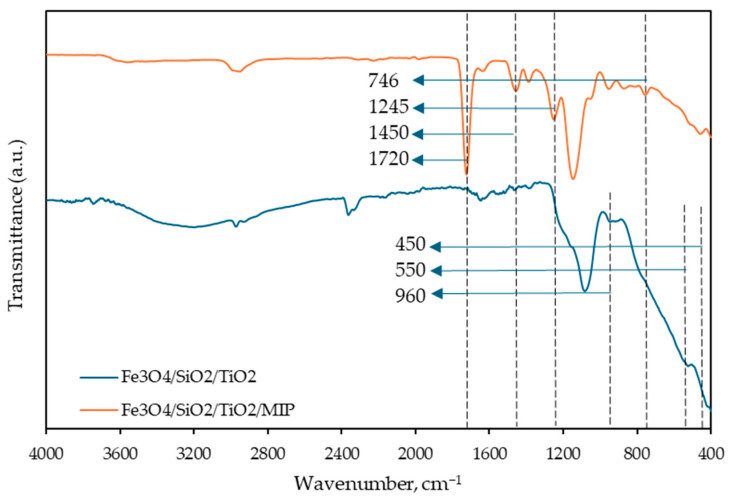
Fourier-transform infrared spectroscopy (FTIR) scans of Fe_3_O_4_/SiO_2_/TiO_2_ (blue line) and Fe_3_O_4_/SiO_2_/TiO_2_/MIP (orange line) nanoparticles.

Figure 3 shows the FTIR spectra for both Fe_3_O_4_/SiO_2_/TiO_2_ and Fe_3_O_4_/SiO_2_/TiO_2_/MIP.

The primary band for magnetite nanoparticles was observed near 550 cm^−1^, corresponding to the Fe–O stretching vibration [32]. A band in the 400–450 cm^−1^ range was attributed to the Ti–O–Ti stretching vibration [33]. The absorption bands between 2800–3800 cm^−1^ were assigned to the stretching vibrations of OH groups, likely from adsorbed water and OH groups bonded to Si and Ti (Si–OH and Ti–OH). Additionally, a small peak around 960 cm^−1^ was identified as the Si–O–Ti stretching vibration, indicating a bond between silica and titanium dioxide layers [34]. As for Fe_3_O_4_/SiO_2_/TiO_2_/MIP nanoparticles, characteristic bands for diclofenac were observed: C−Cl stretching vibration at 746 cm^−1^, C−OH stretching vibration at 1200 cm^−1^, C=C stretching vibration from the aromatic ring at 1450 cm^−1^ and C=O stretching from the carboxyl group at 1720 cm^−1^ [35,36].

**Figure 4:**
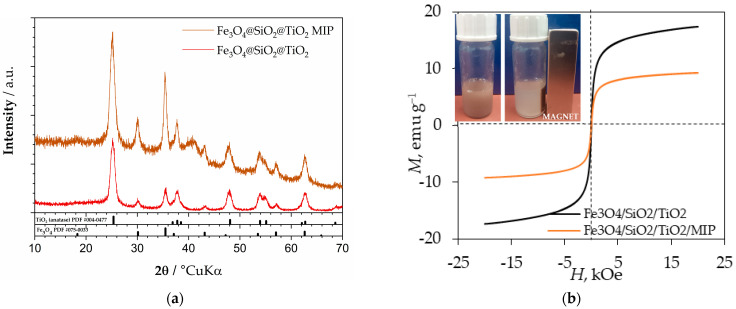
(**a**) Diffractogram of Fe_3_O_4_/SiO_2_/TiO_2_ and Fe_3_O_4_/SiO_2_/TiO_2_/MIP nanoparticles and (**b**) magnetization curves at room temperature of Fe_3_O_4_/SiO_2_/TiO_2_ and Fe_3_O_4_/SiO_2_/TiO_2_/MIP nanoparticles.

Figure 4a shows the diffractogram of the Fe_3_O_4_/SiO_2_/TiO_2_ and Fe_3_O_4_/SiO_2_/TiO_2_/MIP nanoparticles, while Figure 4b shows the magnetization measurements conducted at room temperature. The XRD analysis shows anatase as the main phase (ICDD PDF#21−1272) and magnetite as the minor phase (Fe_3_O_4_) (ICDD PDF#19−0629). A barely visible hump centred around 2θ of 26° characteristic of amorphous silica was evidenced, confirming the presence of the SiO_2_ phase (ICDD PDF# 12-0708).

According to the magnetization curves, the obtained Fe_3_O_4_/SiO_2_/TiO_2_ nanoparticles showed superparamagnetic behaviour with a saturation magnetization value of 17 emu g^−1^ [37,38]. The acquired value of saturation magnetization for Fe_3_O_4_/SiO_2_/TiO_2_/MIP nanoparticles was 9 emu g^−1^. The reduction in saturation magnetization was due to a small increase in the size and weight of the particles after polymerization; however, it was still enough to remove particles from the suspension by applying an external magnetic field [39].

**Figure 5:**
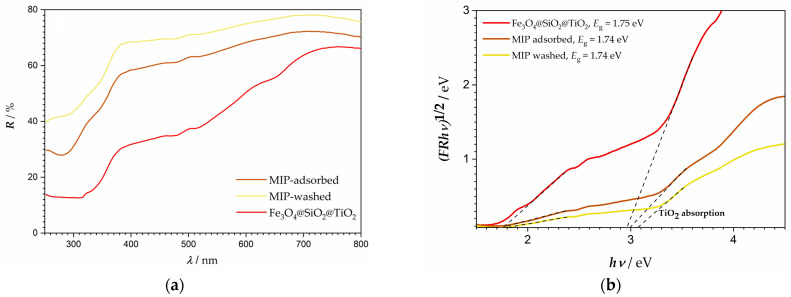
(**a**) The reflectance spectra and (**b**) Tauc’s graphical representation of the prepared molecularly imprinted core–shell photocatalyst.

Figure 5 presents the reflectance spectra as well as Tauc’s graphical representation for the synthesized materials. In complex composite systems, such as these, where multiple semiconductors are contributing to absorption, the lack of a sharp absorption edge can often make the analysis difficult. However, two absorption edges can be observed for the synthesized samples, one at around 1.7 eV, which can be identified as the optical bandgap of Fe_3_O_4_, as is also suggested in literature [40], while the absorption edge at ~3 eV corresponds to titanium dioxides’ excitation [41]. The photoactive catalyst in the system is titanium dioxide, whose spectral range is increased by the broadened absorption spectra of the core–shell nanoparticles. Iron oxide is active in the visible part of the spectra and due to a geometry beneficial for electron transport (core–shell structure), the electrons excited in iron oxide below 3 eV can reach titanium dioxide, thus making it active at energies below 3 eV, that is, wavelengths that belong to the visible range of the spectra.

**Figure 6:**
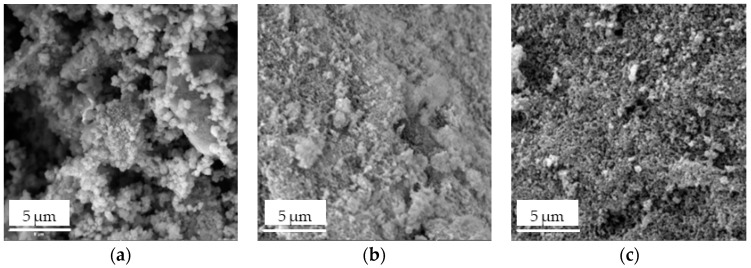
SEM images of (**a**) Fe_3_O_4_/SiO_2_/TiO_2_, (**b**) Fe_3_O_4_/SiO_2_/TiO_2_/MIP adsorbed, and (**c**) Fe_3_O_4_/SiO_2_/TiO_2_/MIP washed nanoparticles.

SEM analysis was used for the investigation of the texture and morphology of the prepared Fe_3_O_4_/SiO_2_/TiO_2_ and Fe_3_O_4_/SiO_2_/TiO_2_/MIP nanoparticles. The obtained SEM images are shown in Figure 6.

The Fe_3_O_4_/SiO_2_/TiO_2_ nanocomposite (Figure 6a) seemed to have a porous microstructure with round particles, which can enhance photocatalytic efficiency. However, the presence of agglomerates might reduce the active surface. To address agglomeration issues, the use of a surfactant and a longer microwave irradiation with high stirring rates may be beneficial. The Fe_3_O_4_/SiO_2_/TiO_2_/MIP adsorbed (Figure 6c) surface became rougher, suggesting the successful synthesis of the polymer imprinted layer. The porosity of the washed Fe_3_O_4_/SiO_2_/TiO_2_/MIP (Figure 6b) seemed to be increased due to the removal of the template molecule.

**Figure 7:**
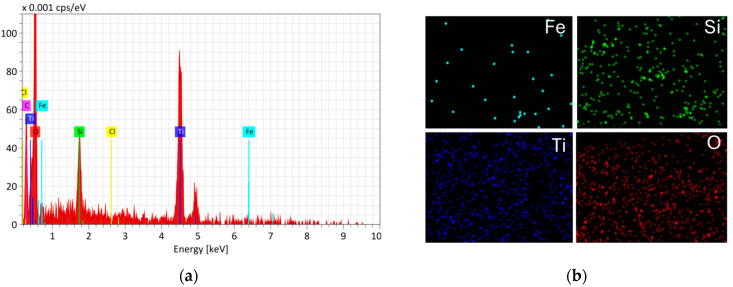
(**a**) EDX spectra and (**b**) corresponding element mapping (Fe, Si, Ti, O) of Fe_3_O_4_/SiO_2_/TiO_2_ nanocomposite.

**Figure 8:**
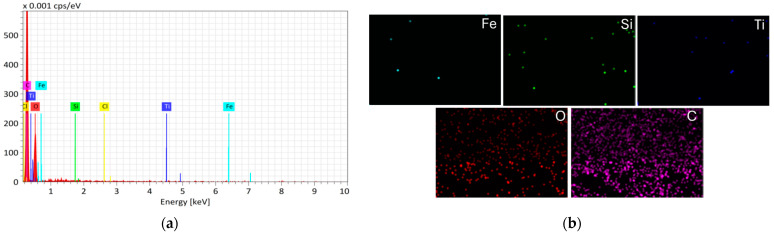
(**a**) EDX spectra and (**b**) corresponding element mapping (Fe, Si, Ti, O and C) of Fe_3_O_4_/SiO_2_/TiO_2_/MIP nanocomposite.

These observations are additionally confirmed by elemental mapping (Figure 7 and Figure 8).

The elemental mapping analysis confirmed the presence of Fe, Si, O, and Ti on the surface of Fe_3_O_4_/SiO_2_/TiO_2_ nanoparticles. Oxygen exhibited the highest concentration, followed by titanium, silicon, and the lowest presence of iron. This distribution is consistent with the core–shell structural model, where Fe constitutes the core, Si forms the intermediate shell, and Ti represents the outermost shell.

The EDX analysis of the Fe_3_O_4_/SiO_2_/TiO_2_/MIP nanocomposite reveals a high concentration of carbon, which is attributed to the presence of the polymer layer. The intensity of peaks for Fe, Si and Ti is quite low due to the polymer coating, which once again confirms the successful polymerization and diclofenac imprinting on core–shell Fe_3_O_4_/SiO_2_/TiO_2_ nanocomposite.

**Figure 9:**
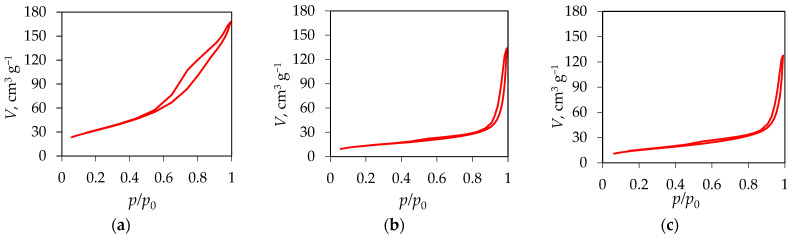
Nitrogen adsorption–desorption isotherms for (**a**) Fe_3_O_4_/SiO_2_/TiO_2_, (**b**) Fe_3_O_4_/SiO_2_/TiO_2_/MIP adsorbed, and (**c**) Fe_3_O_4_/SiO_2_/TiO_2_/MIP washed nanoparticles.

Figure 9 shows the nitrogen adsorption–desorption isotherms for Fe_3_O_4_/SiO_2_/TiO_2_, and adsorbed and washed Fe_3_O_4_/SiO_2_/TiO_2_/MIP. According to the updated IUPAC classification [42], there are eight types of adsorption isotherms, six of which were already identified in the 1985 IUPAC Manual on Reporting Physisorption Data for Gas/Solid Systems. The isotherm obtained for Fe_3_O_4_/SiO_2_/TiO_2_ (Figure 8a) belongs to type IV(a), which is specific to mesoporous materials where the presence of hysteresis is associated with capillary condensation and evaporation within the mesopores [42]. This phenomenon occurs when the pore width exceeds ~4 nm [43,44,45], which is the case for the Fe_3_O_4_/SiO_2_/TiO_2_ sample, as shown in Table 2. On the other hand, two Fe_3_O_4_/SiO_2_/TiO_2_/MIP samples, adsorbed and washed, showed no change in isotherm type (Figure 9b,c), which was predominantly similar to type III, consistent with a non-porous or macroporous solid. However, the small hysteresis loop could be of type H3, which is characteristic of the pore network in a material consisting of macropores that are not completely filled with pore condensate. Even so, it is more likely that the Fe_3_O_4_/SiO_2_/TiO_2_/MIP samples, adsorbed and washed, are macroporous and the isotherms can be treated as reversible.

**Figure 10:**
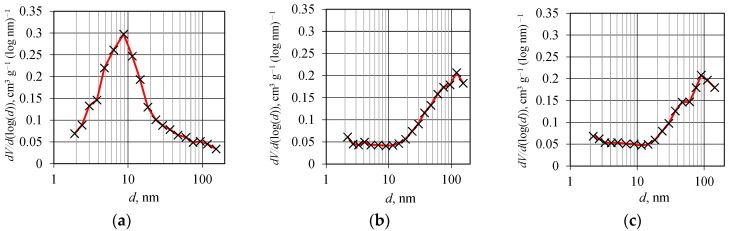
Pore distribution curves of (**a**) Fe_3_O_4_/SiO_2_/TiO_2_, (**b**) Fe_3_O_4_/SiO_2_/TiO_2_/MIP adsorbed, and (**c**) Fe_3_O_4_/SiO_2_/TiO_2_/MIP washed.

Figure 10 shows pore distribution curves of Fe_3_O_4_/SiO_2_/TiO_2_, and adsorbed and washed Fe_3_O_4_/SiO_2_/TiO_2_/MIP. The pore size distribution (Figure 10a–c) confirms a notable shift from the mesoporous (Figure 10a) to the macroporous (Figure 10b,c) region by the addition of a molecularly imprinted layer.

The specific surface area (Table 2) is consistent with the results obtained, which show that as the specific surface area decreases, and the average pore diameter increases with the addition of a molecularly imprinted layer. There was no evident difference between the washed and the adsorbed Fe_3_O_4_/SiO_2_/TiO_2_/MIP samples.

### 3.2. Adsorption Kinetic Studies

**Figure 11:**
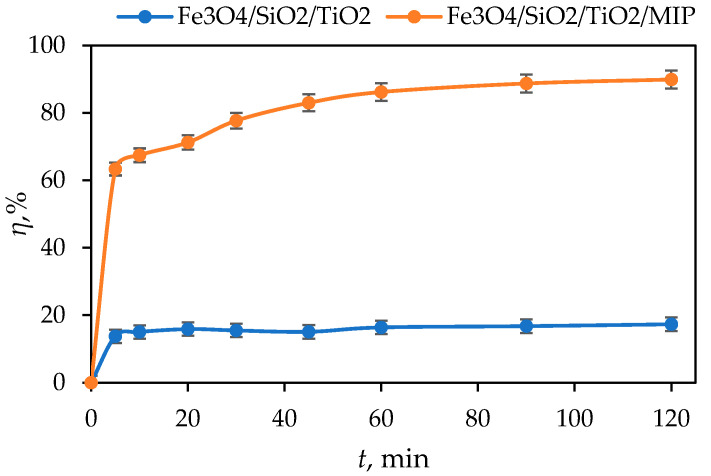
Adsorption efficiency (*η*, %) of diclofenac (DIC) by Fe_3_O_4_/SiO_2_/TiO_2_ and Fe_3_O_4_/SiO_2_/TiO_2_/MIP nanoparticles as a function of time.

Prior to photocatalytic evaluation, adsorption and photolysis tests were performed. The adsorption test was conducted by stirring 100 cm^3^ of the aqueous DIC solution (10 mg dm^−3^) with 50 mg of the Fe_3_O_4_/SiO_2_/TiO_2_/MIP nanoparticles in the dark for 120 min. The results are shown in Figure 11 and were subsequently used to assess the adsorption kinetics. Additionally, the results of the photolysis test showed that diclofenac does not succumb to photolysis under UV-A and SSL irradiation.

The adsorption efficiency was calculated using the following Equation:

(1) $$\eta ,\;\%=\frac{C_{0}-C_{t}}{C_{0}}\times 100,$$

where *η* is the percentage of adsorption efficiency, *C*_0_ (mg dm^−3^) is the initial DIC concentration before adsorption, and *C_t_* (mg dm^−3^) is the concentration of DIC at time *t* (min) of adsorption. The adsorption/desorption equilibrium was established within 60 min with 86% of DIC adsorbed on the surface of Fe_3_O_4_/SiO_2_/TiO_2_/MIP nanoparticles. Given the high percentage of adsorption, the adsorption process kinetics and mechanisms were analysed. On the other hand, the adsorption of DIC on the surface of Fe_3_O_4_/SiO_2_/TiO_2_ nanoparticles was only 16% after 120 min; therefore, the adsorption process kinetics and mechanisms were not analysed in this case. The adsorption capacity, *q* (mg g^−1^) was calculated for Fe_3_O_4_/SiO_2_/TiO_2_ and Fe_3_O_4_/SiO_2_/TiO_2_/MIP according to the following Equation:

(2) $$q=\frac{c_{i}-c_{f}}{m}\times V,$$

where *c_i_* and *c_f_* are the initial and final concentration of DIC (mg dm^−3^), respectively. *V* is the volume of DIC solution (dm^3^), and *m* is the amount of photocatalyst (g) [46].

In addition, the imprinting factor, α, was also calculated according to the following equation:

(3) $$\alpha =\frac{q_{MIP}}{q_{NIP}},$$

where α is the imprinting factor, and *q_MIP_* and *q_NIP_* represent the adsorption capacities of the molecularly imprinted and non-imprinted photocatalyst at equilibrium (60 min) (mg g^−1^) [47,48].

The obtained value of imprinted factor amounts to 4.79. Summarizing the results, it can be concluded that MIP showed a much better binding capacity towards DIC in comparison with the photocatalyst without imprinting. Fe_3_O_4_/SiO_2_/TiO_2_/MIP is characterized as a more selective material with specifically formed sites which allow for a better sorption of DIC according to their shape, size, and interactions between functional groups. To gain deeper insights into the underlying adsorption mechanisms, the adsorption kinetics data were analysed using various model equations, including the Lagergren pseudo-first-order model, the Ho’s pseudo-second-order kinetic model, the Weber–Morris intraparticle diffusion model, and the Boyd diffusion model, as detailed in the following sections [49].

The Lagergren pseudo-first-order model [50] is described by the following Equation:

(4) $$\frac{dq_{t}}{dt}=k_{1}\left(q_{e}-q_{t}\right),$$

where *q_e_* and *q_t_* are the amounts of DIC (mg g^−1^) adsorbed on the Fe_3_O_4_/SiO_2_/TiO_2_/MIP nanocomposite at equilibrium and at time *t*, respectively, while *k*_1_ (min^−1^) is the rate constant of the pseudo-first-order sorption. After integration with the initial conditions of *q =* 0 when *t* = 0, Equation (4) takes the following form:

(5) $$q=q_{e}(1-\exp(-kt)).$$

It can be written in linearized form as follows:

(6) $$\ln\left(q_{e}-q_{t}\right)=lnq_{e}-k_{1}t.$$

**Figure 12:**
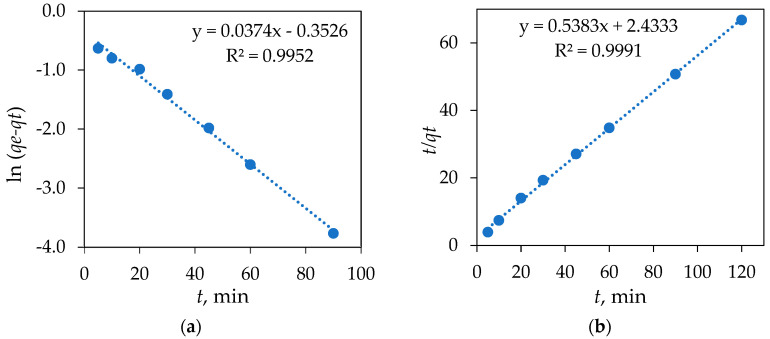
(**a**) Pseudo-first- and (**b**) pseudo-second-order plot of the adsorption of DIC on Fe_3_O_4_/SiO_2_/TiO_2_/MIP nanoparticles.

According to Equation (6), a graph (Figure 12a) of ln (*q_e_* − *q_t_*) against *t* was plotted and obtained *q_e_* = exp(intercept) and *k*_1_ = −(slope).

Furthermore, the kinetic data were analysed using Ho’s pseudo-second-order kinetic model [51], presented as the following equation:

(7) $$\frac{dq_{t}}{dt}=k_{2}{\left(q_{e}-q_{t}\right)}^{2},$$

where *q_e_* and *q_t_* are the amounts of DIC (mg g^−1^) adsorbed on the Fe_3_O_4_/SiO_2_/TiO_2_/MIP nanocomposite at equilibrium and at time *t*, respectively, while *k*_2_ is the rate constant of pseudo-second-order adsorption (g mg ^−1^ min^−1^). By integrating Equation (7) with the initial conditions *q* = 0 at *t* = 0, the linearized form is derived as follows:

(8) $$\frac{1}{q_{t}}=\frac{1}{k_{2}q_{e}^{2}}+\frac{t}{q_{e}}.$$

According to Equation (8), a graph (Figure 12b) of *t*/*q_t_* against 1/*q_e_* was plotted and obtained 1/*k*_2_*q*^2^_*e*_ (intercept) and 1/*q*_*e*_ (slope).

The pseudo-first-order and pseudo-second-order sorption models constants of DIC on Fe_3_O_4_/SiO_2_/TiO_2_/MIP nanoparticles are shown in Table 3. As presented in Table 3, the correlation coefficient for the pseudo-second-order is higher than the correlation coefficient for the pseudo-first-order kinetic model. Moreover, the experimental value *q_e_*_,*exp*_ is quite close to the theoretical value *q_e_*_,*cal*_ for the pseudo-second-order kinetic model. These results indicate that the adsorption of DIC on Fe_3_O_4_/SiO_2_/TiO_2_/MIP nanoparticles might be controlled by the second-order model, with chemisorption likely serving as the rate-limiting step in the adsorption process [52].

### 3.3. Intraparticle Diffusion Model

**Figure 13:**
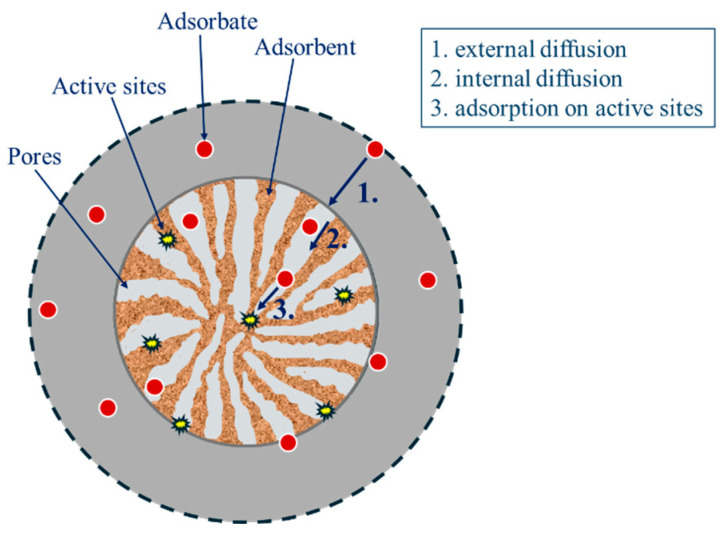
The mechanism of diclofenac adsorption process by Fe_3_O_4_/SiO_2_/TiO_2_/MIP nanoparticles.

Adsorption is the process of the mass transfer of adsorbate (in this case DIC) from the liquid phase to the solid adsorbent (Fe_3_O_4_/SiO_2_/TiO_2_/MIP nanoparticles). The adsorption mass transfer kinetic includes three steps: (1) external mass transfer (film diffusion), (2) intraparticle diffusion, and (3) adsorption on active sites, as shown in Figure 13. It is expected that either film diffusion, intraparticle diffusion, or both can act as rate-limiting steps of DIC adsorption by Fe_3_O_4_/SiO_2_/TiO_2_/MIP nanoparticles.

**Figure 14:**
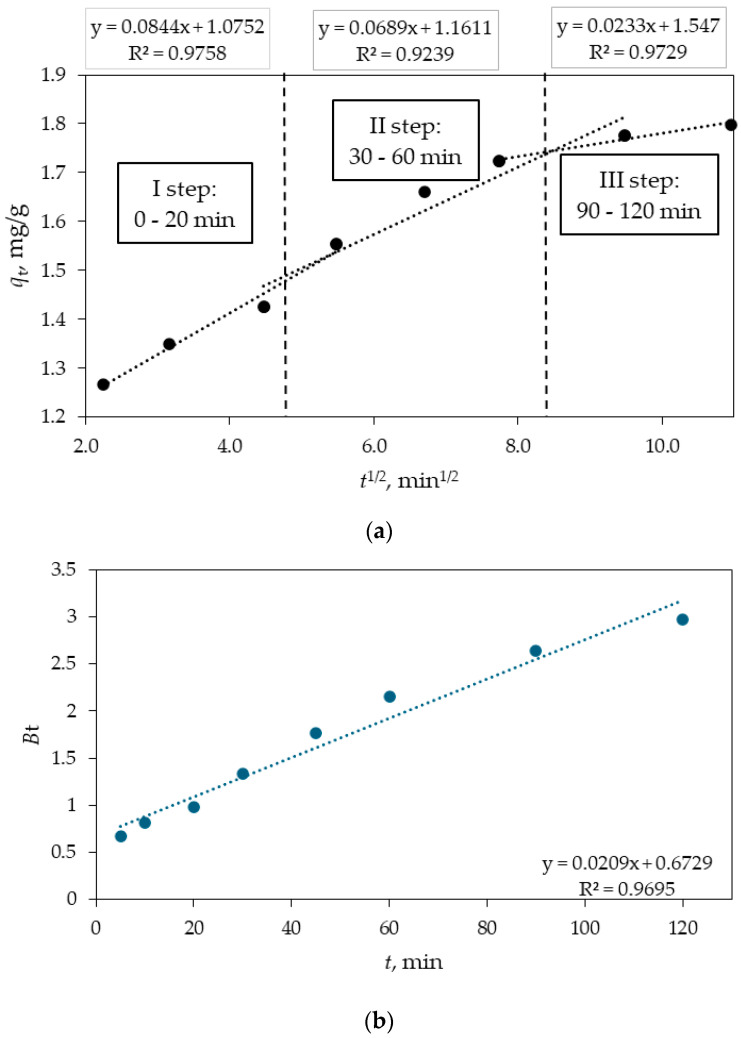
The adsorption kinetic data of DIC on Fe_3_O_4_/SiO_2_/TiO_2_/MIP nanoparticles fitted with (**a**) Webber–Morris intraparticle diffusion and (**b**) Boyd diffusion model.

In order to identify the adsorption mechanism more conclusively and determine the rate-controlling step in the sorption process, the intraparticle diffusion model proposed by Weber and Morris [49] was employed in this study. The equation for the intraparticle diffusion model is expressed as follows:

(9) $$q_{t}=k_{pi}\sqrt{t}+C_{i},$$

where $k_{pi}$ (mg g^−1^ min^−1/2^) is the intraparticle diffusion rate constant and is obtained from the slope of plot $q_{t}$ versus *t*^1/2^, with the intercept being *C_i_*. If intraparticle diffusion is the dominant mechanism, a plot of $q_{t}$ versus *t*^1/2^ will yield a straight line. If this line passes through the origin, it indicates that intraparticle diffusion is the sole rate-controlling step. However, if the plot does not pass through the origin, it suggests the involvement of additional mechanisms alongside intraparticle diffusion. In an adsorption process involving adsorbate and adsorbent, the transfer of the adsorbate may occur via external mass transfer (film diffusion), intraparticle diffusion, or a combination of both mechanisms. Figure 14 shows plot of $q_{t}$ versus *t*^1/2^ consisting of three linear sections with different slopes indicating three different steps taking place during the sorption process.

The sorption process can be divided into three distinct stages: (i) the initial stage with a large slope representing the transport of the adsorbate (DIC) from the bulk solution to the external surface of the adsorbent (Fe_3_O_4_/SiO_2_/TiO_2_/MIP) through film diffusion; (ii) the intermediate stage representing gradual adsorption and corresponding to the diffusion of the adsorbate into the pores of the adsorbent—the transport occurs from the external surface to the interior of the Fe_3_O_4_/SiO_2_/TiO_2_/MIP nanocomposite and is referred to as intraparticle diffusion or inner diffusion—and the (iii) final stage with a small slope representing equilibrium and indicating the system reaching equilibrium. This stage is very fast and cannot be considered as the rate-controlling step. Table 4 lists the model parameters obtained from the plots of the three stages of the sorption process.

Based on the parameters listed in Table 4, we can conclude that the plotted curves do not cross the origin, thus the intraparticle diffusion is not the only rate-controlling step of the sorption process, which is in accordance with the literature findings [53,54,55]. The Boyd diagram, *B*_t_ vs. *t*, was employed to determine the role of film diffusion and intraparticle diffusion in the sorption of DIC [56]. If the plot *B*_t_ vs. *t* crosses the origin and has a regression coefficient higher than 0.99 it could be said that the mechanism controlling the sorption process is intraparticle diffusion. However, since the plot does not pass through its origin and the regression coefficient is less than 0.99, the rate-controlling steps are both intraparticle diffusion and film diffusion, which is consistent with the literature findings [57,58].

In addition to adsorption kinetics and mechanisms, the Langmuir and Freundlich isotherm models were also used to describe the adsorption mechanism [59]. The Langmuir isotherm suggests that the adsorption is monolayer and can be described with the following equation:

(10) $$q_{e}=\frac{Q_{m}K_{L}C_{e}}{1+K_{L}C_{e}},$$

where *q*_*e*_ is the adsorbed amount at equilibrium, mg g^−1^; *Q*_*m*_ is the maximum amount of adsorbed surfactant, mg g^−1^; *K_L_* is the Langmuir constant, dm^3^ mg^−1^; and *C*_*e*_ is adsorbate equilibrium concentration, mg dm^−3^.

The linearized form of Equation (10) is as follows:

(11) $$\frac{1}{q_{e}}=\frac{1}{Q_{m}K_{L}C_{e}}+\left(\frac{1}{Q_{m}}\right).$$

According to Equation (11), a plot of 1/*q_e_* versus 1/*C_e_* is generated and shown in Figure 14a.

To better understand the type and favourability of adsorption, the separation parameter *R_L_* was calculated for the Langmuir model:

(12) $$R_{L}=\frac{1}{1+K_{L}+C_{0}}.$$

If *R_L_* > 1, the adsorption is favourable; if *R_L_*~0, the adsorption is irreversible; if *R_L_* = 1, the adsorption isotherm is linear; and if *R_L_* > 1, the adsorption is unfavourable [60].

The Freundlich isotherm suggests multilayer adsorption on the heterogeneous surface and can be described as follows:

(13) qe=KFCe1n,

where *K_f_* is the sorption efficiency in dm^3^ mg^−1^ and 1/*n* is the adsorption intensity. If 1/*n* < 0.5, the adsorption process is facile; if 1/*n* > 1, the adsorption is cooperative [61].

The linearized form of Equation (13) can be written as follows:

(14) $$logq_{e}=logK_{F}+\frac{1}{n}logC_{e}.$$

**Figure 15:**
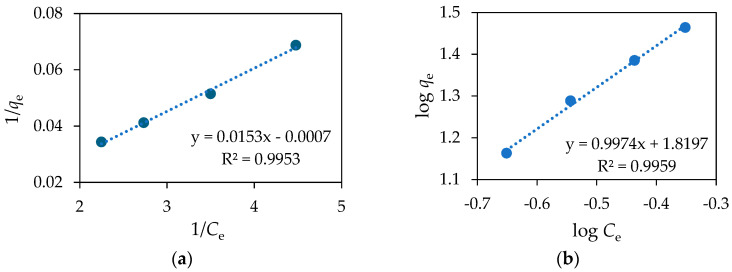
(**a**) Langmuir and (**b**) Freundlich isotherms for the adsorption of DIC on Fe_3_O_4_/SiO_2_/TiO_2_/MIP nanoparticles.

According to Equation (14), a plot of log *q_e_* versus log *C_e_* is generated and shown in Figure 15b. Figure 15 shows the Langmuir and Freundlich isotherms while Table 5 lists the results of the fitted models.

The results show that the correlation coefficients for the Langmuir and Freundlich models are quite close, 0.9953 and 0.9959, respectively. Therefore, it is difficult to say whether the adsorption followed a monolayer or multilayer adsorption behaviour [62,63,64].

The very low value of *R_L_* (0.005) indicates strong adsorbate–adsorbent interactions with a high preference for monolayer adsorption. The low *R_L_* value also suggests that the most favourable sites are occupied, which is consistent with the monolayer adsorption behaviour of the Langmuir model. The value of 1/*n* in the Freundlich model is 1.00, which suggests minimal surface homogeneity, thus further supporting the Langmuir model even though the correlation coefficient is slightly higher for the Freundlich model than for the Langmuir model.

### 3.4. Photocatalytic Studies

**Figure 16:**
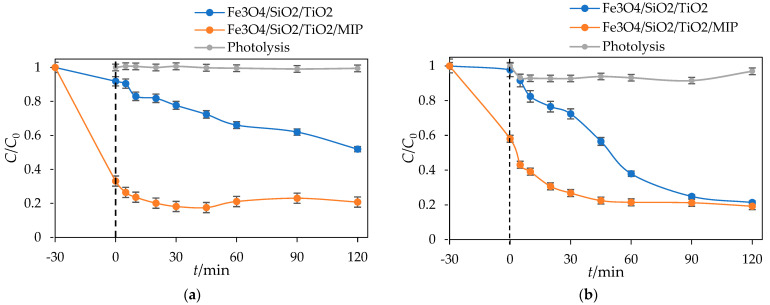
Photolysis and photocatalytic degradation of DIC by Fe_3_O_4_/SiO_2_/TiO_2_ and Fe_3_O_4_/SiO_2_/TiO_2_/MIP nanoparticles as a function of irradiation time under (**a**) UV lamp and (**b**) sun simulator lamp.

Figure 16 presents the photocatalytic degradation of DIC by Fe_3_O_4_/SiO_2_/TiO_2_ and Fe_3_O_4_/SiO_2_/TiO_2_/MIP nanoparticles under UV-A and sun simulator irradiation.

It can be seen from Figure 16a that the concentration of DIC was lowered by around 50% after 30 min adsorption and 120 min of UV-A irradiation by Fe_3_O_4_/SiO_2_/TiO_2_ nanoparticles. On the other hand, the concentration of DIC was lowered by around 80% after 30 min adsorption and 120 min of UV-A irradiation by Fe_3_O_4_/SiO_2_/TiO_2_/MIP nanoparticles. As for solar-simulated light, (Figure 16b), the concentration of DIC was lowered by around 80% after 30 min adsorption and 120 min SSL by both Fe_3_O_4_/SiO_2_/TiO_2_ and Fe_3_O_4_/SiO_2_/TiO_2_/MIP.

**Figure 17:**
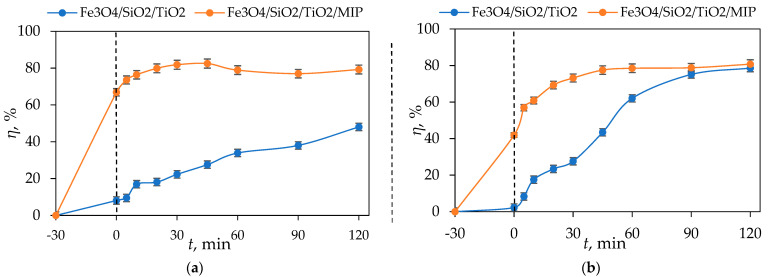
The degradation efficiency of DIC by Fe_3_O_4_/SiO_2_/TiO_2_ and Fe_3_O_4_/SiO_2_/TiO_2_/MIP nanoparticles under (**a**) UV lamp and (**b**) sun simulator lamp.

The removal efficiency of DIC by Fe_3_O_4_/SiO_2_/TiO_2_ and Fe_3_O_4_/SiO_2_/TiO_2_/MIP nanoparticles after adsorption and UV-A- and solar-simulated irradiation was calculated according to Equation (1) and is shown in Figure 17.

Table 6 provides a comparative overview of various photocatalysts used for the degradation of DIC by TiO_2_-based photocatalysts. For comparison, the results obtained in this study are added at the bottom of the table.

TiO_2_ (P25) MIP shows moderate degradation under UV light, whereas unmodified TiO_2_ (P25) reaches 80% in combination with H_2_O_2_. TiO_2_-based zeolite achieves the highest efficiency (90%) under solar-simulated light. AC-TiO_2_-N reaches 80% of degradation with a UV-immersed lamp, while TiO_2_ achieves only 50% with a UV-immersed lamp. The newly developed Fe_3_O_4_/SiO_2_/TiO_2_/MIP achieves 80% degradation under both UV light and SSL, indicating competitive or better performance than the referenced systems. This improved photocatalytic performance is due to the core–shell structure which enhances charge separation and reduces electron–hole recombination.

Table 7 presents the sorption capacity of DIC on various sorbents, as well as the sorption capacity of DIC on prepared Fe_3_O_4_/SiO_2_/TiO_2_/MIP photocatalyst, for comparison.

It can be seen from Table 6 that the prepared Fe_3_O_4_/SiO_2_/TiO_2_/MIP photocatalyst exhibited a sorption capacity of 1.86 mg g^−1^ at a DIC concentration of 10 mg dm^−3^. Even though this value is relatively lower than other sorbents in this table (due to the limited number of imprinted sites), it is important to point out that this material is designed to function as a photocatalyst, not only as an adsorbent. This synergistic effect of adsorption and photocatalysis might show the enhanced performance of the prepared photocatalyst for the selective removal of pharmaceuticals (in this case DIC) from water. Future investigation will involve the use of other pharmaceuticals as well as their mixtures. Moreover, other parameters affecting adsorption and photocatalysis will be investigated, such as pH, temperature, and concentration of pharmaceuticals, as well as the amount of photocatalyst used.

For the photocatalytic degradation of DIC, the kinetic constants (*k*) were calculated by the following equations for the pseudo-first-order model:

(15) $$-ln\left(\frac{C_{t}}{C_{0}}\right)=k_{1}t,$$

and pseudo-second-order model:

(16) $$\frac{1}{C_{t}}-\frac{1}{C_{0}}=k_{2}t,$$

where *C_t_* is the concentration at a specific time (mg dm^−3^), *C*_0_ is the initial concentration (mg dm^−3^), and *t* is time (min).

Table 8 presents the pseudo-first-order and pseudo-second-order kinetic constants (*k*_1_, *k*_2_) for the photocatalytic degradation of DIC under UV- and sun-simulated irradiation.

According to Table 8, kinetic constants are higher for sun-simulated irradiation for both Fe_3_O_4_/SiO_2_/TiO_2_ and Fe_3_O_4_/SiO_2_/TiO_2_/MIP nanoparticles. The correlation coefficients for the pseudo-first order model are higher than those for the pseudo-second-order model, indicating that the photocatalytic process is controlled by the pseudo-first-order model.

### 3.5. Scavenger Experiments

**Figure 18:**
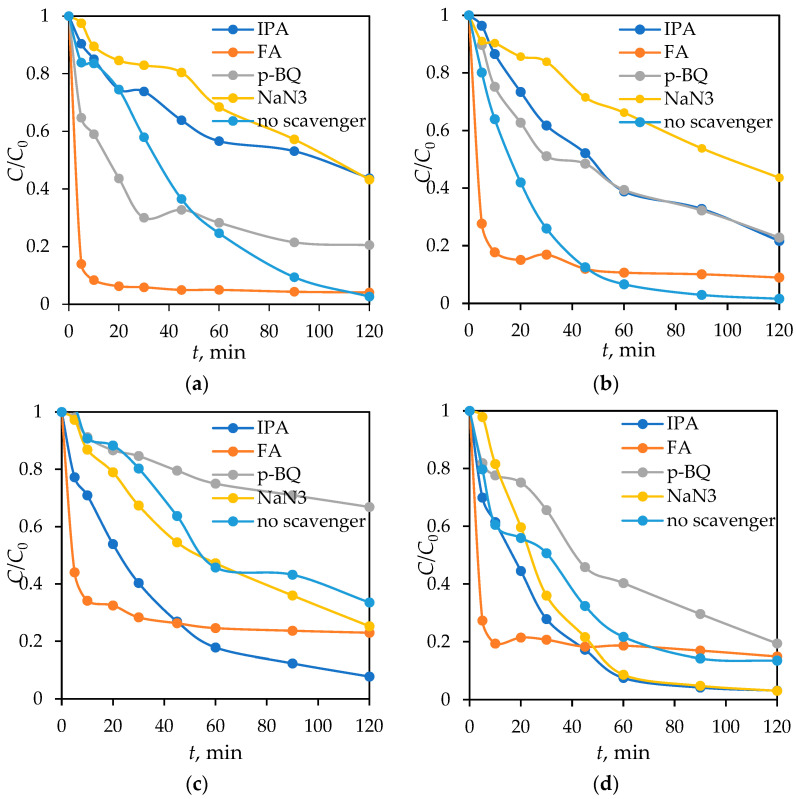
DIC degradation by Fe_3_O_4_/SiO_2_/TiO_2_/MIP under (**a**) UV- and (**b**) solar-simulated light and Fe_3_O_4_/SiO_2_/TiO_2_ nanoparticles under (**c**) UV- and (**d**) solar-simulated light with the addition of scavengers.

Four types of scavengers were used: isopropanol (IPA) as a quencher of hydroxyl radicals (^●^OH), formic acid (FA) for holes (*h*^+^), p-benzoquinone (p-BQ) for superoxide radicals (O_2_^●−^), and sodium azide (NaN_3_) as a singlet oxygen (^1^O_2_) scavenger [76,77,78]. Figure 18 shows the photocatalytic degradation of DIC by Fe_3_O_4_/SiO_2_/TiO_2_ and Fe_3_O_4_/SiO_2_/TiO_2_/MIP nanoparticles with the addition of the aforementioned scavengers under UV- and solar-simulated light.

It can be seen on Figure 18 that DIC is very susceptible to different reactive oxygen species (ROS) scavengers. The obtained results demonstrate that the presence of sodium azide significantly influences the degradation of DIC when using Fe_3_O_4_/SiO_2_/TiO_2_/MIP as a photocatalyst under both UV light and SSL. This suggests that singlet oxygen (^1^O_2_) plays a crucial role in the degradation mechanism. Additionally, under UV light, isopropanol exhibits a similar effect as NaN_3_, indicating that hydroxyl radicals (•OH) also contribute to DIC degradation. Therefore, the degradation process by Fe_3_O_4_/SiO_2_/TiO_2_/MIP is primarily driven by singlet oxygen under SSL, whereas both singlet oxygen and hydroxyl radicals are responsible under UV light.

In contrast, when using Fe_3_O_4_/SiO_2_/TiO_2_ nanoparticles without a molecularly imprinted template, the inhibiting order or examined scavengers was p-BQ > formic acid > azide > IPA, while under SSL, photogenerated holes also exhibit a notable impact. These results suggest that Fe_3_O_4_/SiO_2_/TiO_2_ nanoparticles facilitate DIC degradation through different pathways depending on the light source: primarily superoxide radicals under UV light, and a combination of superoxide radicals and holes under SSL.

Also, a notably higher kinetic rate of DIC degradation is observed after the addition of hydroxyl and superoxide radical scavengers compared to the reaction without a scavenger. By incorporating two materials such as TiO_2_ and Fe_2_O_3_ in a new photocatalyst, the formation of a heterojunction can increase the photocatalytic activity, considering the light source used. This material interface can reduce electron–hole pair recombination and increase pharmaceutical degradation by the IPA absorption of ^●^OH radicals and photogenerated holes, since alcohols can act as both ^●^OH and photogenerated hole scavengers [79,80,81]. The addition of formic acid also accelerated photocatalysis, which is in agreement with previously published work, describing in detail the mechanism of radical species transfer through an iron and TiO_2_ lattice causing the same trend of improved degradation after the addition of a hole scavenger. The main contribution of both superoxide radicals and holes in DIC photocatalysis are in agreement with a previously published paper where a g−C_3_N_4_ photocatalyst was used [82]. All experiments followed a pseudo-first kinetic order; the kinetic parameters are summarized in Table 9.

## 4. Conclusions

This work presents the successful preparation of Fe_3_O_4_/SiO_2_/TiO_2_/MIP nanoparticles by microwave-assisted synthesis and polymerization.

- The obtained particles were analysed via FTIR and XRD, which confirmed the successful synthesis of Fe_3_O_4_/SiO_2_/TiO_2_/MIP nanoparticles.
- Vibrating-sample magnetometry showed the superparamagnetic behaviour of the prepared samples.
- SEM images showed the increased surface roughness of the Fe_3_O_4_/SiO_2_/TiO_2_/MIP nanoparticles due to the synthesis of the imprinted polymer layer, while the washed Fe_3_O_4_/SiO_2_/TiO_2_/MIP nanoparticles exhibited higher porosity due to the removal of the template molecule. Elemental mapping confirmed the core–shell structure and successful molecular imprinting.
- The bandgap amounts to 1.7 eV, which can be identified as the optical bandgap of Fe_3_O_4_ and ~3 eV, which corresponds to titanium dioxides’ excitation.
- The Fe_3_O_4_/SiO_2_/TiO_2_ nanoparticles have the highest specific surface area (116.6 m^2^ g^−1^) but the lowest average pore diameter (8.5 nm), whereas adsorbed Fe_3_O_4_/SiO_2_/TiO_2_/MIP has the lowest specific surface area (49.7 m^2^ g^−1^) but highest average pore diameter (12.3 nm).
- The adsorption of DIC by Fe_3_O_4_/SiO_2_/TiO_2_ was only 16% after 120 min while the photocatalytic degradation by Fe_3_O_4_/SiO_2_/TiO_2_ nanoparticles under UV-A- and solar-simulated irradiation was around 40% and 80% after 120 min, respectively.
- The synergistic removal of DIC by Fe_3_O_4_/SiO_2_/TiO_2_/MIP achieved an adsorption of around 86% after 60 min, and a photocatalytic degradation of around 80% after 120 min of both UV-A and solar-simulated irradiation.
- The adsorption kinetic results indicate that the adsorption of DIC on Fe_3_O_4_/SiO_2_/TiO_2_/MIP nanoparticles could be controlled by the second-order model. The intraparticle diffusion model alongside the Boyd diagram suggest the rate controlling steps are both intraparticle diffusion and film diffusion.
- Fe_3_O_4_/SiO_2_/TiO_2_/MIP demonstrates a degradation mechanism predominantly driven by singlet oxygen under SSL and a combination of singlet oxygen and hydroxyl radicals under UV light, while the Fe_3_O_4_/SiO_2_/TiO_2_ nanoparticles rely on superoxide radicals under UV light and a combination of superoxide radicals and photogenerated holes under SSL.

These results demonstrate that the combination of MIP with Fe_3_O_4_/SiO_2_/TiO_2_ improves the adsorption efficiency and photocatalytic degradation, making Fe_3_O_4_/SiO_2_/TiO_2_/MIP nanoparticles a promising material for the selective removal of DIC and potentially other pollutants. The magnetic properties allow for easy separation and the possibility of reuse.

## Figures and Tables

**Table 1:** Scavengers of reactive oxygen species (ROSs).

Scavengers	Reactive Oxygen Species (ROSs)
isopropanol (IPA)	hydroxyl radicals (^●^OH)
formic acid (FA)	holes (*h*^+^)
*p*-benzoquinone (p-BQ)	superoxide radicals (O_2_^●−^)
sodium azide (NaN_3_)	singlet oxygen (^1^O_2_)

**Table 2:** Specific surface area, average pore diameter and pore volume of prepared Fe_3_O_4_/SiO_2_/TiO_2_, and adsorbed and washed Fe_3_O_4_/SiO_2_/TiO_2_/MIP.

Material	Specific Surface Area	Average Pore Diameter	Pore Volume
	*S*_BET_, m^2^ g^−1^	*d*_avg_, nm	*V*_pore_, cm^3^ g^−1^
Fe_3_O_4_/SiO_2_/TiO_2_	116.6	8.5	0.2591
Fe_3_O_4_/SiO_2_/TiO_2_/MIP adsorbed	49.7	12.3	0.1820
Fe_3_O_4_/SiO_2_/TiO_2_/MIP washed	55.7	11.4	0.1858

**Table 3:** The pseudo-first-order and pseudo-second-order sorption models constants of DIC on Fe_3_O_4_/SiO_2_/TiO_2_/MIP nanoparticles.

	Kinetic Model					
Pseudo-First Order				Pseudo-Second Order		
*q_e_*_,*exp*_, mg g^−1^	*k*_1_, min^−1^	*q_e_*_,*cal*_, mg g^−1^	*R* ^2^	*k*_2_, g mg^−1^ min^−1^	*q_e_*_,*cal*_, mg g^−1^	*R* ^2^
1.80	0.04	0.70	0.9952	0.12	1.86	0.9991

**Table 4:** Intraparticle diffusion model constants and correlation coefficients for the adsorption of DIC on Fe_3_O_4_/SiO_2_/TiO_2_/MIP nanoparticles.

Intraparticle Diffusion								
First Stage of Sorption			Second Stage of Sorption			Third Stage of Sorption		
*k*p_1_, mg g^−1^ min^−1/2^	*C*_1_	*R* ^2^	*k*p_2_, mg g^−1^ min^−1/2^	*C*_1_	*R* ^2^	*k*p_3_, mg g^−1^ min^−1/2^	*C*_3_	*R* ^2^
0.084	1.07	0.9758	0.069	1.16	0.9239	0.023	1.55	0.9729

**Table 5:** Langmuir and Freundlich model constants and correlation coefficients for the adsorption of DIC on Fe_3_O_4_/SiO_2_/TiO_2_/MIP nanoparticles.

Langmuir Model				Freundlich Model		
*Q_m_*, mg g^−1^	*K_L_*, dm^−3^ mg^−1^	*R* ^2^	1/*n*	*K_F_*, mg g^−1^	*R* ^2^	*R_L_*
65.36	21.86	0.9953	1.00	66.02	0.9959	0.005

**Table 6:** Degradation efficiency of DIC removal by TiO_2_-based photocatalysts.

Photocatalyst	Light Source	Degradation Efficiency, %	Ref.
TiO_2_ (P25) MIP	UV	60	[6]
TiO_2_ (P25) MIP	UV	70	[15]
TiO_2_ (P25)	UV (4 × 2.78 W m^−2^) UV/H_2_O_2_	20 80	[65]
TiO_2_-based zeolite	SSL (1247.8 W m^−2^)	90	[66]
AC-TiO_2_-N	UV-immersed lamp	80	[67]
TiO_2_	UV-immersed lamp	50	[68]
Fe_3_O_4_/SiO_2_/TiO_2_/MIP	UV (98.5 W m^−2^) SSL (1028.6 W m^−2^)	80	This work

**Table 7:** Sorption capacity of DIC on different sorbents.

Sorbent	Diclofenac Concentration, mg dm^−3^	Sorption Capacity, mg g^−1^	Ref.
Reduced graphene oxide (rGO)	40	22.64	[69]
Granular activated carbon	5 100	35 150	[70]
Organobentonite with surfactant hexadecyltrimethylammonium	600	125–250	[71]
Hexagonal mesoporous silicate	0.1	0.032	[72]
Powdered activated carbon	5	4.47	[73]
Thermo-plasma expanded graphite	100	400	[74]
Einkorn husk activated carbon	50	147.06	[75]
Fe_3_O_4_/SiO_2_/TiO_2_/MIP	10	1.86	This work

**Table 8:** The values of pseudo-first and pseudo-second order kinetic constants (*k*_1_, *k*_2_) and correlation coefficients (*R*^2^) of DIC removal by Fe_3_O_4_/SiO_2_/TiO_2_ and Fe_3_O_4_/SiO_2_/TiO_2_/MIP nanoparticles under UV- and sun-simulated irradiation.

Material		Pseudo-First Order		Pseudo-Second Order	
	Lamp	*k*_1_, min^−1^	*R* ^2^	*k*_2_, dm^3^ mg^−1^ min^−1^	*R* ^2^
Fe_3_O_4_/SiO_2_/TiO_2_	UV	0.0044	0.9799	0.0204	0.9797
	SSL	0.0137	0.9695	0.1546	0.9646
Fe_3_O_4_/SiO_2_/TiO_2_/MIP	UV	0.0027	0.9515	0.0982	0.9245
	SSL	0.013	0.9273	0.126	0.8258

**Table 9:** Pseudo-first order kinetic constant (*k*_1_), half-life time (*t*_1/2_), and determination coefficient (*R*^2^) of scavengers.

Material		UV			SSL		
		*k*_1_, min^−1^	*t*_1/2_, min	*R* ^2^	*k*_1_, min^−1^	*t*_1/2_, min	*R* ^2^
Fe_3_O_4_/SiO_2_/TiO_2_	IPA	0.0213	32.54	0.9718	0.0302	22.95	0.9533
	FA	0.0065	106.64	0.8452	0.0051	135.91	0.8445
	p-BQ	0.0033	210.04	0.9349	0.0131	52.91	0.9861
	NaN_3_	0.0115	60.27	0.9933	0.0319	21.73	0.9656
	No scavenger	0.0098	70.73	0.9560	0.0173	40.07	0.9785
Fe_3_O_4_/SiO_2_/TiO_2_/MIP	IPA	0.0064	108.30	0.9490	0.0127	54.58	0.9829
	FA	0.0100	69.31	0.8129	0.0115	60.27	0.8906
	p-BQ	0.0115	60.27	0.7831	0.0115	60.27	0.9530
	NaN_3_	0.0066	105.02	0.9776	0.0067	103.45	0.9925
	No scavenger	0.0562	12.33	0.9773	0.0456	15.20	0.9995

## Data Availability

The original contributions presented in this study are included in the article/Supplementary Material. Further inquiries can be directed to the corresponding authors.

## Supplementary Materials

The following supporting information can be downloaded at https://www.mdpi.com/article/10.3390/ma18102300/s1. Figure S1: Inner pressure, temperature, and power supplied by the microwave oven during the synthesis of Fe_3_O_4_ nanoparticles; Figure S2: Inner pressure, temperature, and power supplied by the microwave oven during the synthesis of Fe_3_O_4_/SiO_2_/TiO_2_ nanocomposite; Figure S3: Inner pressure, temperature, and power supplied by the microwave oven during the synthesis of Fe_3_O_4_/SiO_2_/TiO_2_/MIP; Figure S4: Schematic representation of photocatalytic mechanism for DIC on Fe_3_O_4_/SiO_2_/TiO_2_/MIP; Figure S5: UV-VIS absorption spectra of diclofenac over time during (a) adsorption on Fe_3_O_4_/SiO_2_/TiO_2_, (b) adsorption on Fe_3_O_4_/SiO_2_/TiO_2_/MIP, (c) photocatalysis with UV-A lamp and Fe_3_O_4_/SiO_2_/TiO_2_, (d) photocatalysis with UV-A lamp and Fe_3_O_4_/SiO_2_/TiO_2_/MIP, (e) photocatalysis with SSL lamp and Fe_3_O_4_/SiO_2_/TiO_2_ and (f) photocatalysis with SSL lamp and Fe_3_O_4_/SiO_2_/TiO_2_/MIP; Figure S6. Zeta potential measurements for Fe_3_O_4_/SiO_2_/TiO_2_ nanoparticles and Figure S7: Effect of contact time on adsorption of various concentrations of DIC on Fe_3_O_4_/SiO_2_/TiO_2_/MIP.
