# Supplementary material for: Synergistic Removal of Diclofenac via Adsorption and Photocatalysis Using a Molecularly Imprinted Core–Shell Photocatalyst
Source: Materials (Basel). 2025 May 15;18(10):2300. doi: 10.3390/ma18102300 (PMC12113530; doi:10.3390/ma18102300)
Supplement: Supplementary file 1 [file materials-18-02300-s001.zip › materials-3608192-supplementary.pdf]

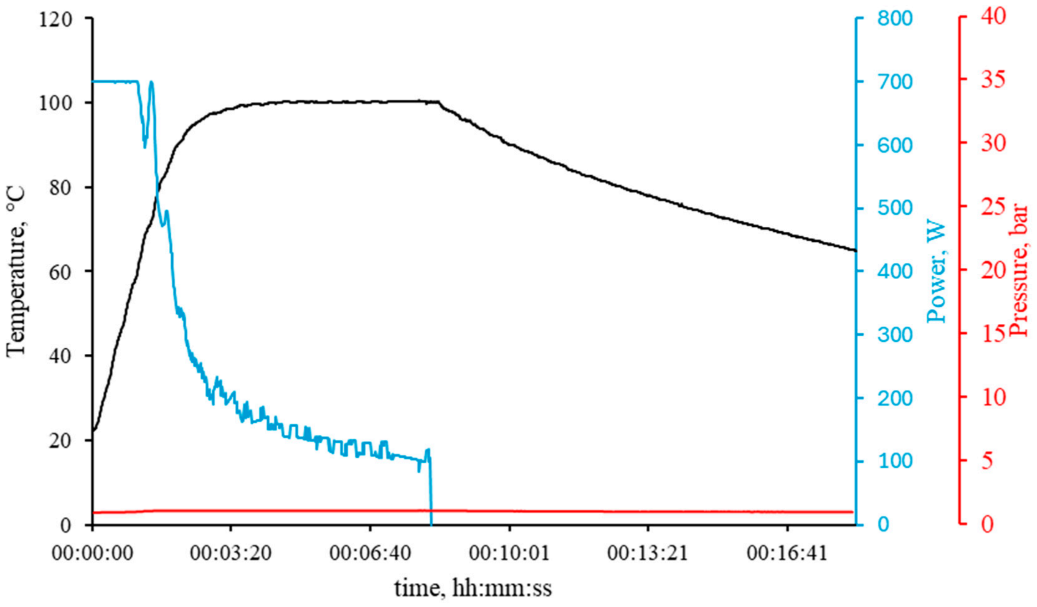

Figure S1. Inner pressure, temperature, and power supplied by the Microwave oven during the synthesis of Fe_3O_4 nanoparticles.

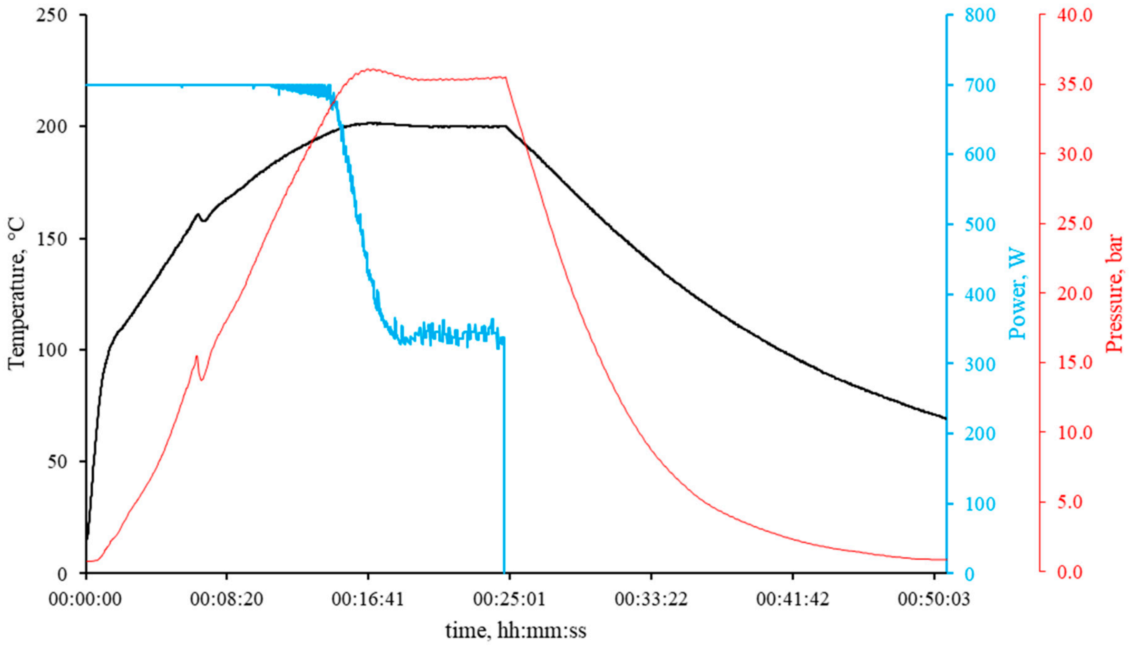

Figure S2. Inner pressure, temperature, and power supplied by the Microwave oven during the synthesis of $Fe_3O_4/SiO_2/TiO_2$ nanocomposite.

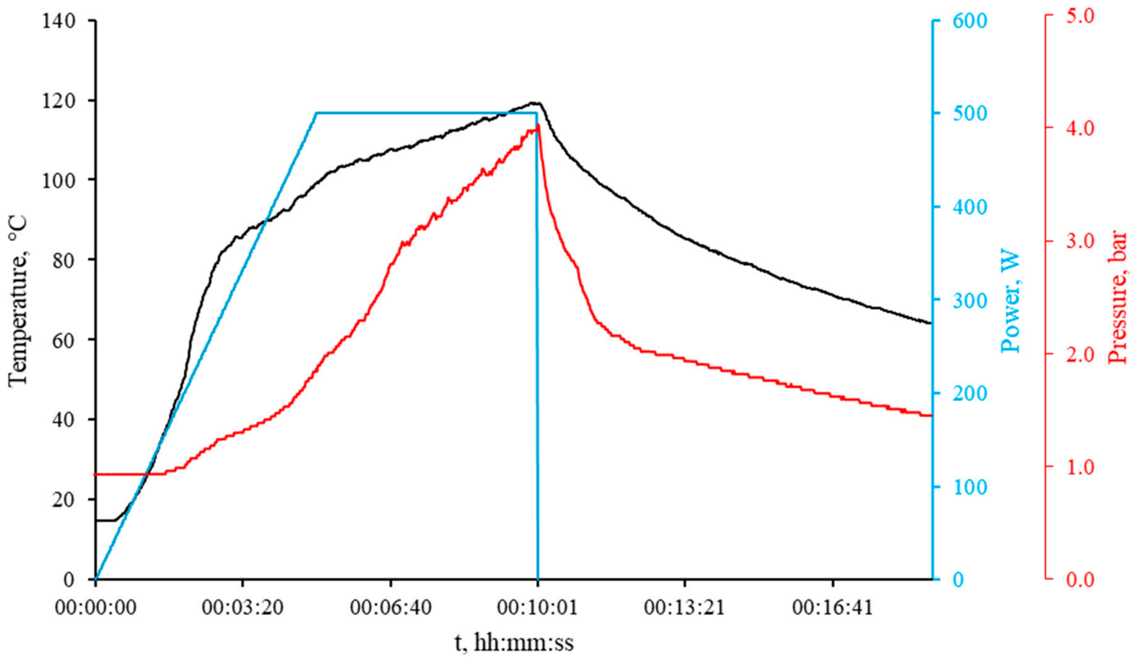

Figure S3. Inner pressure, temperature, and power supplied by the Microwave oven during the synthesis of $Fe_3O_4/SiO_2/TiO_2$/MIP.

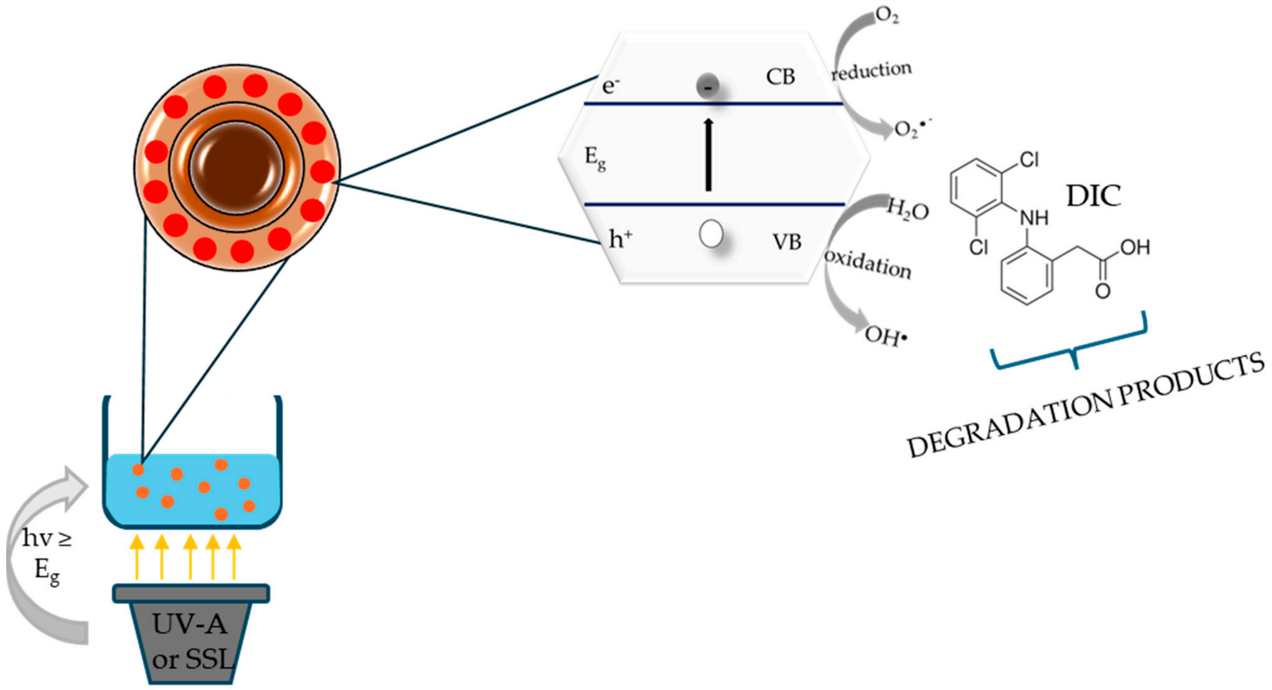

Figure S4. Schematic representation of photocatalytic mechanism for DIC on $Fe_3O_4/SiO_2/TiO_2$/MIP.

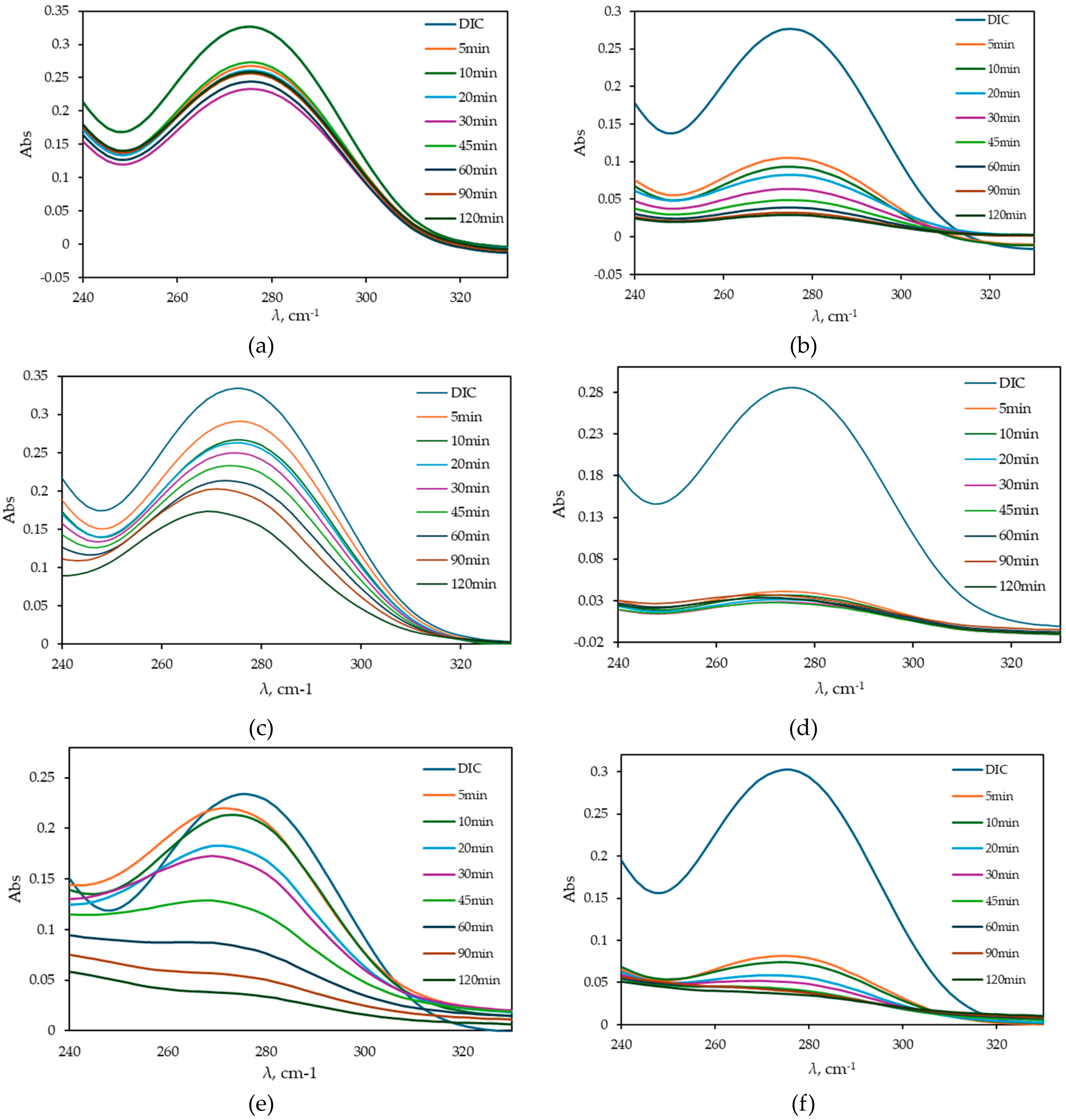

Figure S5. UV-VIS absorption spectra of diclofenac over time during (**a**) adsorption on $Fe_3O_4/SiO_2/TiO_2$, (**b**) adsorption on $Fe_3O_4/SiO_2/TiO_2$/MIP, (**c**) photocatalysis with UV-A lamp and $Fe_3O_4/SiO_2/TiO_2$, (**d**) photocatalysis with UV-A lamp and $Fe_3O_4/SiO_2/TiO_2$/MIP, (**e**) photocatalysis with SSL lamp and $Fe_3O_4/SiO_2/TiO_2$ and (**f**) photocatalysis with SSL lamp and $Fe_3O_4/SiO_2/TiO_2$/MIP

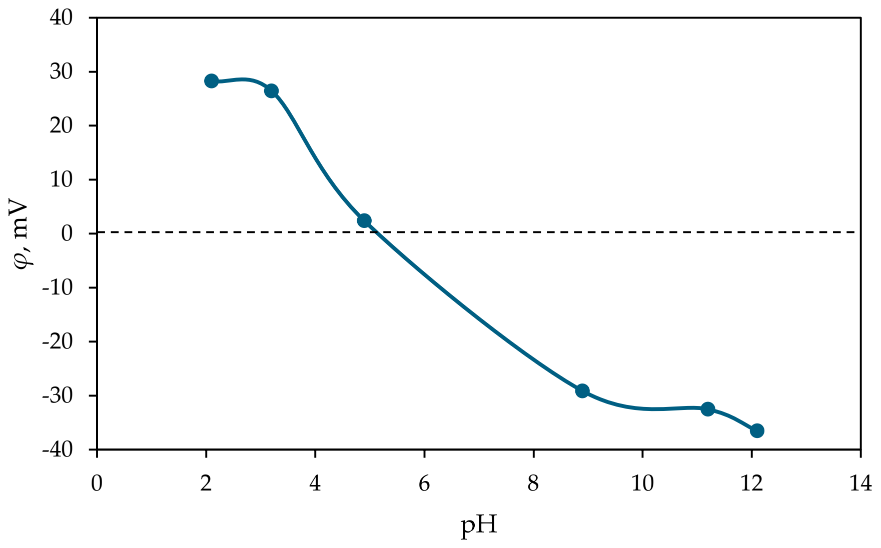

Figure S6. Zeta potential measurements for $Fe_3O_4/SiO_2/TiO_2$ nanoparticles.

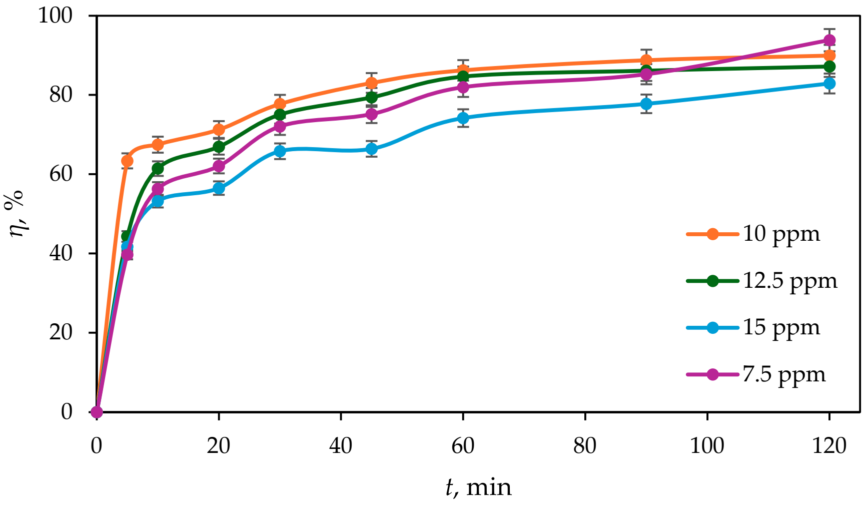

Figure S7. Effect of contact time on adsorption of various concentrations of DIC on $Fe_3O_4/SiO_2/TiO_2$/MIP.
